# Case Report: Thoracic SMARCA4-deficient undifferentiated tumor with extraocular muscle metastasis: a rare case hidden in diplopia

**DOI:** 10.3389/fmed.2026.1877658

**Published:** 2026-07-09

**Authors:** Bingying Liu, Xuan Wang, Yongqing Liu, Jue Wang, Wanzhen Jiao

**Affiliations:** 1 Department of Ophthalmology, Shandong Provincial Hospital Affiliated to Shandong First Medical University, Jinan, Shandong, China; 2 Department of Ophthalmology, Shandong Second Provincial General Hospital, Jinan, Shandong, China

**Keywords:** case report, diagnosis, extraocular muscle metastasis, lung neoplasm, SMARCA4-UT

## Abstract

**Background:**

Thoracic SMARCA4-deficient undifferentiated tumor (SMARCA4-UT) is a recently recognized, highly aggressive malignancy characterized by loss of SMARCA4 (BRG1) expression and a strong propensity for early metastasis. Orbital metastases are rare, and involvement of extraocular muscles (EOMs) has not been previously reported.

**Case presentation:**

A 60-year-old man presented with a 5-day history of binocular diplopia and significant ocular pain. Ophthalmic examination revealed mild left eye proptosis, exotropia, and restricted ocular motility. Orbital imaging demonstrated a nodular lesion involving the left inferior rectus muscle, initially suspected to be an orbital inflammatory pseudotumor. Despite systemic steroid therapy, symptoms progressed. Surgical excision of the lesion revealed an undifferentiated tumor with cystic–solid features. Immunohistochemistry showed positivity for cytokeratin and INI-1, complete loss of BRG1 expression, a high Ki-67 index, and wild-type p53, consistent with SMARCA4-deficient undifferentiated tumor. Subsequent systemic evaluation identified a primary lesion in the left upper lung lobe with mediastinal lymphadenopathy and adrenal gland involvement. Mediastinal lymph node biopsy confirmed metastatic SMARCA4-UT. The patient died soon after one cycle of chemotherapy and immunotherapy, with brain metastases as the likely cause of death.

**Conclusion:**

This case represents the first reported instance of thoracic SMARCA4-UT presenting initially as an isolated metastasis to the inferior rectus muscle. It underscores the diagnostic challenges posed by atypical orbital lesions and highlights the importance of early tissue diagnosis when a suspected inflammatory condition fails to respond to therapy. SMARCA4-UT should be considered in the differential diagnosis of extraocular muscle masses, as early recognition is critical for appropriate oncologic management.

## 1 Introduction

Thoracic SMARCA4-deficient undifferentiated tumors (SMARCA4-UT) represent a highly aggressive class of malignancies, most frequently arising in the thoracic cavity of middle-aged male smokers. First characterized by Le Loarer et al. in 2015 as SMARCA4-deficient thoracic sarcomas (1), this entity was formally listed as a distinct tumor type in the 2021 WHO Classification of Thoracic Tumors (2). Its hallmark features include an undifferentiated or rhabdoid morphology and a complete loss of SMARCA4 (BRG1) protein expression. SMARCA4-UTs are recognized as being highly aggressive, often presenting with local invasion and widespread metastasis at the time of diagnosis (3, 4). Common metastatic sites include lymph nodes, bone, and adrenal glands. Orbital metastases from any primary tumor are rare, accounting for only 2%−7% of orbital masses. Involvement of the extraocular muscles (EOMs) is extremely uncommon (5). Notably, orbital metastasis serves as the initial presentation of an occult malignancy in only 15% of cases (6).

Here, we present a diagnostically challenging case of a thoracic SMARCA4-UT. Its first clinical manifestation was an isolated metastasis to the inferior rectus muscle, a presentation that has not been previously documented.

## 2 Case presentation

A 60-year-old man presented with a 5-day history of binocular diplopia accompanied by significant ocular pain. The patient had a well-documented history of active smoking spanning 40 years, with a daily consumption of approximately one pack of cigarette. Over the past 2 years, the smoking frequency had escalated to two packs per day. He had no significant past medical history and no family history of hereditary diseases or malignancies.

### 2.1 Initial ophthalmic evaluation

On initial ophthalmic examination, uncorrected visual acuity was 6/18 binocular, with the best-corrected visual acuity of 6/6 and 6/10 in the right and left eye, respectively. Intraocular pressure on non-contact tonometry was 13 mm of Hg in both the eyes. Slit-lamp examination and fundus examination was unremarkable in both eyes. Formal orthoptic assessment demonstrated a large-angle left exotropia of more than 45° in primary gaze on Hirschberg test, and prism examination revealed a left exotropia of 90 prism diopters (PD) deviation. The left eye exhibited mild proptosis, complete limitation of upgaze and downgaze, and mild defect in adduction and abduction. It is important to note that Hess chart and diplopia charting could not be obtained, as the patient was unable to cooperate due to severe ocular pain at presentation. This limits the detailed characterization of the diplopia and ocular motility disturbance, though the prism measurements confirmed a significant exotropic deviation.

### 2.2 Imaging and laboratory findings

**Figure 1:**
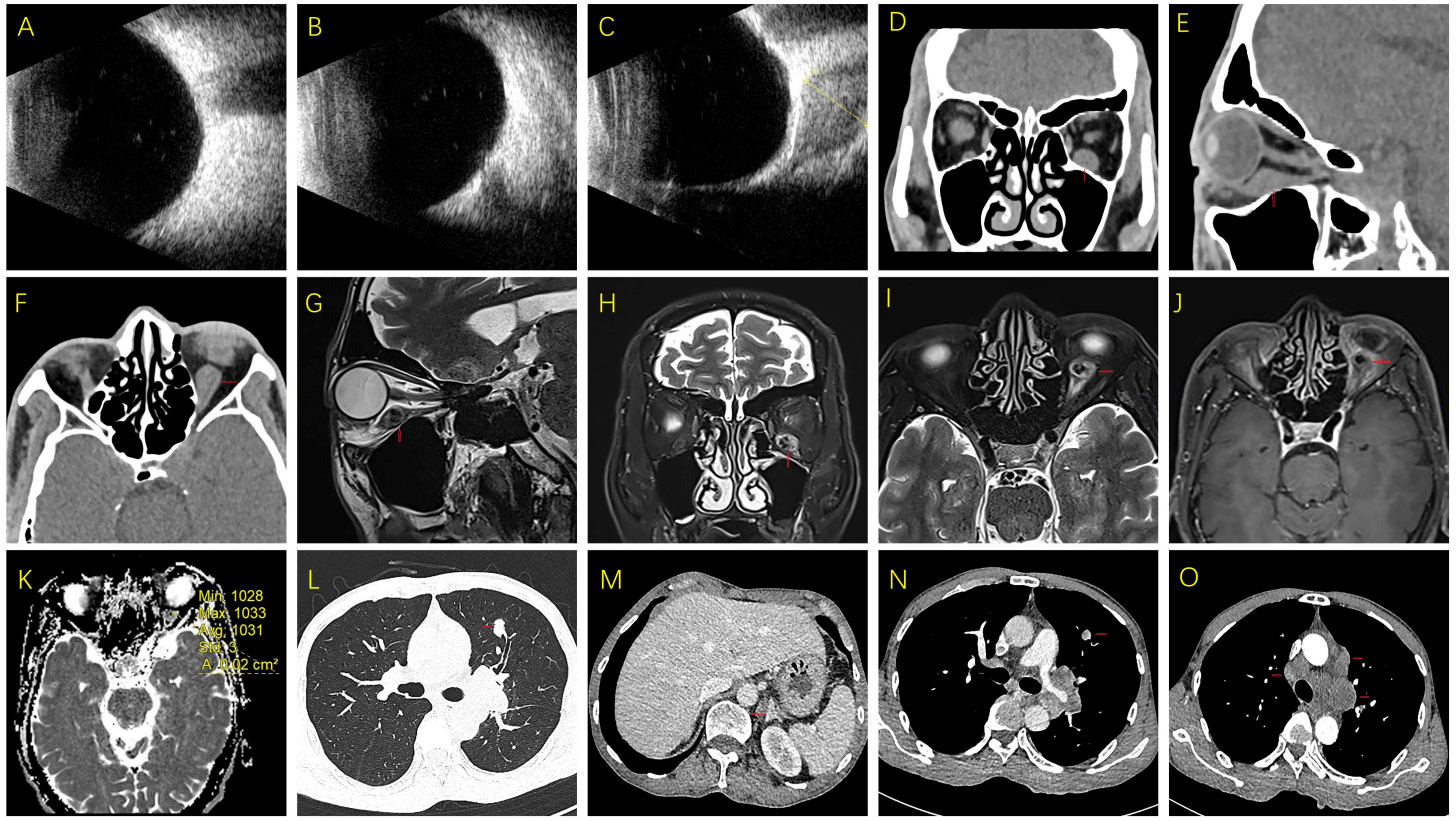
Ophthalmic B-scan ultrasonography, plain orbital CT scan, orbital magnetic resonance imaging, plain chest CT scans, abdominal CT scans, and contrast-enhanced chest CT scan. The images show vitreous opacities in both right **(A)** and left eye **(B)**. A lesion in the left orbit involving the inferior rectus muscle was revealed. The yellow double-headed arrow indicates the measured width of the inferior rectus muscle belly (8.40 mm) **(C)**. **(D–F)** Axial CT images in the soft tissue window demonstrate diffuse thickening of the left inferior rectus muscle, most prominently involving its muscular belly (red arrows). **(G–I)** Axial T2-weighted MRI reveals a well-circumscribed annular hyperintense lesion located within the medial aspect of the left inferior rectus muscle, measuring approximately 1.1 × 0.8 cm, with patchy intralesional hypointensities. The adjacent tendon exhibits swelling and T2 hyperintensity suggestive of edema, while the surrounding orbital fat is preserved without abnormal signals (red arrows). **(J)** Contrast-enhanced T1-weighted MRI shows peripheral ring-like enhancement with a central non-enhancing region (red arrow). **(K)** ADC map demonstrates restricted diffusion, with values ranging from 1.03-1.15 mm^2^/s. **(L)** A soft tissue density nodule in the anterior segment of the left upper lobe with clear boundaries. Lobulated margins, spiculations, and vessel convergence sign are identified at the edge of the nodule (red arrow). **(M)** The left adrenal gland appears thickened (red arrow). **(N)** A markedly enhancing nodule with heterogenous enhancement in the anterior segment of the left upper lobe (red arrow). **(O)** Multiple enlarged lymph nodes in the left hilum and mediastinum (red arrow).

Ophthalmic ultrasonography B-scan revealed marked enlargement of the inferior rectus muscle (Figures 1A–C). A plain orbital computed tomography (CT) scan demonstrated diffuse thickening of the left inferior rectus muscle, most prominent in the muscle belly (Figures 1D–F). On orbital magnetic resonance imaging (MRI), the lesion manifests as a focal muscular mass within the inferior rectus muscle belly, with no evidence of fat infiltration or stranding in the surrounding orbital adipose tissue. The mass does not directly invade the adjacent tendon (Figures 1G–I); however, edema is appreciable in the neighboring tendon on T2-weighted imaging (T2WI). The lesion demonstrates high signal intensity on T2WI. Contrast-enhanced imaging showed heterogeneous enhancement, with a cross-sectional size of approximately 1.1 × 0.8 cm (Figure 1J). The apparent diffusion coefficient (ADC) value ranges from 1.03 to 1.15 mm^2^/s (Figure 1K).

Thyroid and cervical lymph node ultrasonography identified thyroid nodules classified as TI-RADS 3. Laboratory testing revealed normal thyroid function and normal serum IgG4 levels, thereby excluding thyroid-associated orbitopathy and IgG4-related disease. Brain parenchymal MRI and magnetic resonance venography showed no abnormalities. Based on these findings, an initial diagnosis of left orbital inflammatory pseudotumor was made.

### 2.3 Disease progression and re-evaluation

Systemic steroid therapy was initiated after admission but did not show significant clinical improvement. The initial diagnosis of orbital inflammatory pseudotumor was based on a combination of clinical and imaging findings. Clinically, the patient presented with typical features including exotropia, mild proptosis, restricted ocular motility, and significant ocular pain—all of which are consistent with an inflammatory process. Ocular B-scan ultrasonography demonstrated marked thickening of the left inferior rectus muscle with associated tendon swelling, a pattern not confined to the muscle belly and frequently observed in inflammatory conditions such as thyroid-associated orbitopathy or orbital pseudotumor. Notably, the presence of a non-solid, ill-defined lesion within the extraocular muscle did not exclude the possibility of inflammatory changes, as early or atypical inflammatory pseudotumors may present with similar features.

However, when systemic steroid therapy failed to produce clinical improvement, we reassessed the imaging findings. MRI subsequently revealed a well-defined mass with heterogeneous, ring-shaped enhancement—features more suggestive of a neoplastic process than a purely inflammatory lesion. This discordance between the clinical expectation (steroid-responsive inflammation) and the imaging appearance prompted us to pursue surgical exploration.

### 2.4 Surgical intervention and pathological findings

In order to attain a more optimal visualization of the tumor margins, the patient underwent endoscopic resection of the left orbital lesion and extended tumor resection under general anesthesia. Intraoperatively, the mass was located within the belly of the inferior rectus muscle. The necrotic tissue and partial solid components were detected within the cystic area, characterized by ill-defined boundaries, rich blood supply, and a soft texture. Given this high likelihood of malignant metastasis, it was temporarily determined to conduct a wide excision of the tumor while retaining only a minimal quantity of tendon tissue, instead of adhering to the conventional protocol of cryopreservation followed by extensive resection. The lesion was completely excised and submitted for pathological examination, and the fluid was sent for bacterial culture.

**Figure 2:**
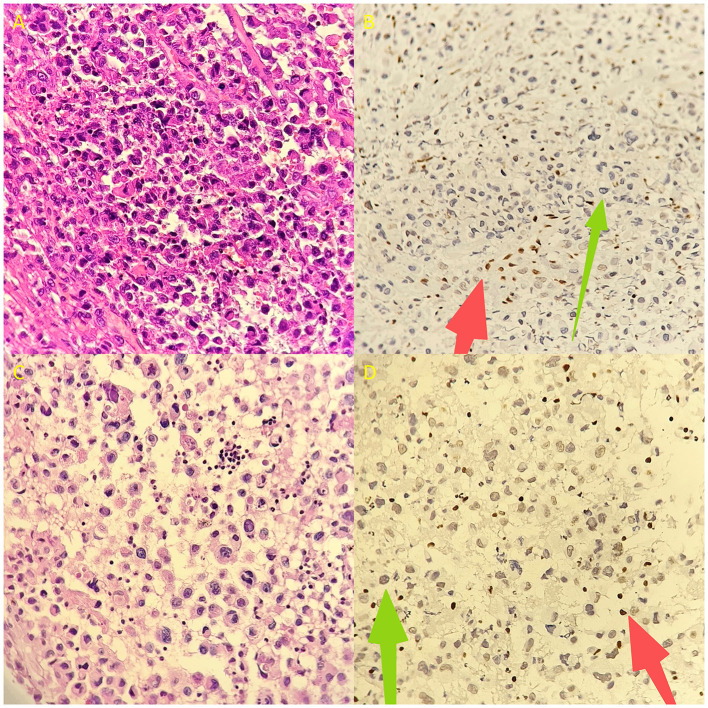
Histologic findings. **(A)** Hematoxylin and eosin staining of the extraocular muscle tumor, showing large tumor cells with eosinophilic cytoplasm and eccentric nuclei. **(B)** Immunohistochemistry staining of the extraocular muscle tumor, revealing loss of BRG1 expression in tumor cells (green arrow), while stromal cells exhibiting positive expression as an internal control (red arrow). **(C)** Hematoxylin and eosin staining of the lung tumor, showing sheets of tumor cells with large cell bodies, eosinophilic cytoplasm and eccentric nuclei. **(D)** Immunohistochemistry staining of the lung tumor, revealing loss of BRG1 expression in tumor cells (green arrow), while stromal cells exhibiting positive expression as an internal control (red arrow).

Histopathological analysis (Figure 2) revealed undifferentiated tumor morphology. Immunohistochemistry showed that tumor cells were positive for cytokeratin (CK) and INI-1, with a complete loss of BRG1 expression. The tumor was negative for CK7, CK20, TF-1, Napsin A, CDX-2, INSM1, CD38, P40, P63, CK5/6, NUT, and HMB45. The p53 staining pattern was wild type, and the Ki-67 proliferation index was approximately 45%. It showed no growth on bacterial culture. These findings supported a diagnosis of SMARCA4-deficient undifferentiated tumor involving the left inferior rectus muscle.

### 2.5 Systemic evaluation and final diagnosis

Postoperatively, the pain had significantly alleviated. Additionally, a further systemic evaluation was conducted to determine whether the extraocular muscle mass is primary or secondary.

Plain computed tomography (CT) scans of the chest and abdomen (Figure 1N) demonstrated a soft tissue nodule within the anterior segment of the left upper lung lobe, with dimensions of approximately 1.6 × 1.1 cm. The nodule exhibited lobulated margins, spiculations, and vessel convergence (Figure 1L). Multiple enlarged lymph nodes were observed in the left hilum and mediastinum, with the largest one measuring approximately 2.9 × 2.7 cm. Thickening of the left adrenal gland was also noted.

Contrast—enhanced chest CT (Figures 1M–O) showed marked enhancement of the pulmonary nodule (approximately 89 HU). Multiple heterogeneously enhancing enlarged lymph nodes were found in the left hilar, mediastinal, and bilateral cervical regions. The left adrenal gland was still thickened, and a hypoenhancing nodule about 1.6 cm in size detected in the right adrenal gland.

Serum tumor marker analysis revealed elevated levels of neuron-specific enolase (NSE, 23.21 ng/mL) and squamous cell carcinoma antigen (SCCA, 3.4 ng/mL). Endobronchial ultrasound-guided transbronchial needle aspiration (EBUS-TBNA) of the mediastinal lymph nodes was subsequently carried out. Pathological examination revealed the presence of malignant tumor cells. Immunohistochemical analysis showed positive staining CK and INI-1 with complete loss of BRG1 expression, and negative staining for CK7, TTF-1, PSA, SYN, S-100, HMB45, P40, CD5, and CD117.

Based on the clinical presentation, imaging findings and histology profiles, the diagnosis of SMARCA4-deficient undifferentiated tumor was confirmed. The Ki-67 index was approximately 60%, and p53 remained wild type.

Therefore, the final diagnosis of this case was metastasis of the left extraocular muscle by primary lung source (SMARCA4-deficient undifferentiated tumor) (Table 1). Unfortunately, the patient was discharged after diagnosis and did not continue care at our institution. Subsequent follow-up revealed that the patient died soon after one cycle of chemotherapy and immunotherapy, with brain metastases as the likely cause of death.

**Table 1:** The clinical course of the patient.

Medical stage	Examinations and interventions	Conclusions
Initial diagnosis	Ophthalmic examination and orbital imaging were performed.	An orbital inflammatory pseudotumor was suspected.
Empirical treatment	Systemic steroid therapy was administered.	Symptoms progressed.
Imaging re-evaluation	Repeated and reassessment of ocular imaging examinations.	Extraocular muscle space-occupying lesion was highly suspected, and surgical exploration was scheduled.
Surgery	The lesion was completely excised and submitted for pathological examination.	Histopathological analysis supported a diagnosis of SMARCA4-deficient undifferentiated tumor.
Systemic evaluation	Contrast-enhanced chest CT, serum tumor marker analysis, EBUS-TBNA of mediastinal lymph nodes were completed to identify tumor origin.	The final diagnosis was metastasis of the left extraocular muscle by primary lung source (SMARCA4-deficient undifferentiated tumor).

CT, computed tomography; EBUS-TBNA, endobronchial ultrasound-guided transbronchial needle aspiration.

## 3 Discussion

SMARCA4-UT is a highly aggressive thoracic malignancy characterized by rapid progression and early, widespread metastasis. It tends to infiltrate adjacent structures such as the pleura, cervical region, and lung apex (1). Common metastatic sites include lymph nodes, bone, and adrenal glands (5), consistent with the present patient. Ocular and orbital metastases are rare, with approximately 100 cases reported (3).

While intraocular metastasis from SMARCA4-UT has been reported recently (7), EOM involvement, as observed in this case, has not been previously described, making this case unique.

Orbital metastases account for 2%−7% of all orbital tumors. EOM involvement is even rarer (8), representing only about 5% of orbital metastases (9), typically affecting a single muscle—most commonly the lateral rectus, followed by the medial rectus, superior rectus, inferior rectus, and oblique muscles (10). Clinically, patients present with proptosis, diplopia, ptosis, and restricted ocular motility (11). Because orbital metastases usually occur via hematogenous dissemination, EOM metastasis generally indicates advanced systemic disease and poor prognosis (12). Breast and lung cancers are the most frequent primary sources (13).

Diagnosis of SMARCA4-UT is challenging due to nonspecific manifestations that mimic inflammatory or neoplastic processes. Accurate diagnosis requires integration of clinical history, imaging, pathology, and immunohistochemistry (4). CT and MRI are primary imaging modalities; CT is often the initial imaging choice, whereas MRI provides superior soft-tissue resolution and better delineation of extraocular muscles and surrounding structures (6, 12). On contrast-enhanced MRI, inflammatory lesions such as orbital inflammatory pseudotumor typically demonstrate relatively homogeneous enhancement (14), whereas malignant tumors, including SMARCA4-UT, more commonly show heterogeneous enhancement. However, imaging alone is insufficient. Failure to respond to steroid therapy should prompt early tissue biopsy.

*SMARCA4* (SWI/SNF-related, matrix-associated, actin-dependent regulator of chromatin, subfamily A, member 4) is a tumor suppressor gene encoding the BRG1, a core component of the SWI/SNF chromatin-remodeling complex. This complex plays a critical role in transcriptional regulation, DNA repair and replication, chromosomal organization, and cellular differentiation. Mutations or deletions of *SMARCA4* have been identified in various aggressive malignancies (15). Loss of BRG1expression is central to diagnosis, with most cases showing absent *BRG1* staining and a high Ki-67 index (16).

Differential diagnosis is crucial, as SMARCA4-UT shares morphological and clinical features with several other entities. In conjunction with the clinical presentation and biopsy features, loss of SMARCA4 (BRG1) expression on immunohistochemistry confirmed the diagnosis of thoracic SMARCA4-UT. But distinguishing thoracic SMARCA4-UT from SMARCA4-deficient non-small cell lung carcinoma (NSCLC) is a critical challenge because both entities show loss of SMARCA4 (BRG1) expression. However, they differ in clinical presentation, morphology, and immunophenotype. SMARCA4-UT typically presents as a large, rapidly growing thoracic mass with frequent necrosis. A study reported primary tumor sizes of SMARCA4-UT ranging from 2.2 to 18.3 cm (mean 9.2 cm) (17). Notably, the lesion in our patient (1.6 cm) falls at the lower end of this spectrum, reflecting the heterogeneity of tumor size at presentation. Additionally, SMARCA4-UT often lacks a recognizable glandular or squamous component on biopsy as shown in our case. In contrast, SMARCA4-deficient NSCLC usually arises in association with conventional NSCLC histology (adenocarcinoma or squamous cell carcinoma) and may show partial retention of epithelial features (18, 19). Furthermore, SMARCA4-UT shows primitive, undifferentiated, or rhabdoid morphology with cohesive sheets of epithelioid cells, while SMARCA4-deficient NSCLC often retains some evidence of lung primary differentiation (e.g., lepidic, acinar, or solid patterns with intracellular mucin). In immunohistochemistry, SMARCA4-UT is typically positive for pan-cytokeratin (often focal or weak), INI-1 (retained), and may express SOX2, but is negative for lung-specific markers such as TTF-1, Napsin A, and CK7. SMARCA4-deficient NSCLC, by comparison, frequently shows stronger and more diffuse CK7 positivity, and may express TTF-1 or p40 depending on its lineage (19, 20). In our case, the orbital and mediastinal tumor cells were negative in CK7, TTF-1, and Napsin A, arguing against a conventional NSCLC origin. Notably, loss of SMARCA4 is often accompanied by retained or upregulated SMARCA2 (BRM) expression in many tumors. SMARCA2 (BRM) is the alternative ATPase subunit of the SWI/SNF complex (21). In SMARCA4-UT, concomitant loss of SMARCA2 expression occurs in a substantial subset (approximately 30%−50% of cases) and is associated with more aggressive behavior (5). Importantly, diffuse loss of both SMARCA4 and SMARCA2 is highly suggestive of SMARCA4-UT rather than SMARCA4-deficient NSCLC, where SMARCA2 expression is more frequently retained (22). Therefore, in diagnostically challenging cases, immunohistochemical assessment of SMARCA2 (BRM) expression can provide additional value. This distinction aids in differentiating these two entities, especially when clinical and morphological features overlap. It should be highlighted that molecular confirmation is not always required for diagnosis when typical IHC findings are present, but it can be crucial in ambiguous cases. Targeted sequencing or whole-exome sequencing can identify SMARCA4 loss-of-function mutations (nonsense, frameshift, splice-site) or homozygous deletions (23). These mutations are present in the vast majority of SMARCA4-UT and SMARCA4-deficient NSCLC, but the latter often harbors additional driver mutations (e.g., KRAS, EGFR, STK11, KEAP1) that are typically absent in SMARCA4-UT (24). Copy number analysis may detect homozygous SMARCA4 deletion, which is more common in SMARCA4-UT than in SMARCA4-deficient NSCLC (23). RNA sequencing or DNA methylation profiling provide emerging evidence that suggests distinct methylation signatures between SMARCA4-UT and other SMARCA4-deficient tumors, though this is not yet standard practice (25, 26). In our case, given the classic IHC profile (complete loss of BRG1, retained INI-1, wild-type p53, high Ki-67) and consistent clinical and radiologic features, the diagnosis of SMARCA4-UT was firmly established without molecular testing. However, we acknowledge that in atypical presentations, molecular testing would be strongly recommended. Additionally, in contrast to malignant rhabdoid tumors, SMARCA4-UT occurs predominantly in middle-aged and elderly patients and is genetically distinct. SMARCA4-UT can be differentiated from small-cell carcinoma of the ovary, hypercalcemic type (SCCOHT), by its immunohistochemical profile, as SCCOHT typically expresses WT1, epithelial membrane antigen, vimentin, and cytokeratin (4).

Management of ocular metastases hinges on identifying the primary tumor, which dictates systemic therapy. Ocular metastasis may be the sentinel event of occult malignancy, as in this case, requiring urgent search for the primary source. As summarized in Supplementary Table S1 (27–46), while a narrow spectrum of carcinomas—notably breast and lung—accounts for the majority of cases, each primary tumor imparts a distinct clinical signature on its ocular deposit.

Systemic control is the cornerstone of therapy; local ocular treatment is adjunctive. For example, a choroidal metastasis from a hormone receptor-positive breast cancer may regress with endocrine therapy alone, whereas melanoma or renal cell carcinoma may require immunotherapy or targeted agents. While external beam radiotherapy (EBRT) remains the workhorse for symptomatic choroidal metastases, offering excellent rates of local control and visual stabilization, its application is nuanced. For a patient with a solitary metastasis from an indolent primary and a favorable systemic prognosis, plaque brachytherapy might be chosen for its precise dose conformity and sparing of surrounding structures. The management of bilateral disease, common in breast cancer, may necessitate modified techniques. Finally, the primary tumor is the principal determinant of prognosis. The outlook for a patient with an ocular metastasis from controlled breast cancer is measured in years, justifying aggressive local sight-preserving therapies. For a patient with newly diagnosed, widespread lung cancer, the prognosis is often measured in months, prompting more focus on quality of life with rapid symptomatic relief. This prognostic awareness is essential for realistic goal-setting with patients and families.

Ocular metastasis should be viewed as part of systemic disease, requiring close collaboration among ophthalmologist, radiologist, pathologist and oncologist. The goal is to harmonize effective local control of the ocular threat with the overarching systemic management of the patient's cancer, ensuring that preservation of vision is thoughtfully integrated into the broader palliative care plan.

## 4 Conclusion

This case highlights the need for high suspicion of malignancy in atypical orbital lesions, especially when clinical and imaging are inconclusive and steroid therapy fails. Accurate identification of SMARCA4-UT is critical, as management differs from inflammatory conditions. This report expands the metastatic spectrum of SMARCA4-UT and underscores its consideration in the differential diagnosis of extraocular muscle lesions.

## Data Availability

The original contributions presented in the study are included in the article/Supplementary material, further inquiries can be directed to the corresponding author/s.
