# Supplementary material for: Case Report: Thoracic SMARCA4-deficient undifferentiated tumor with extraocular muscle metastasis: a rare case hidden in diplopia
Source: Front Med (Lausanne). 2026 Jul 9;13:1877658. doi: 10.3389/fmed.2026.1877658 (PMC13391907; doi:10.3389/fmed.2026.1877658)
Supplement: Supplementary file 1 [file Table_1.docx]

**Table S1.** Ocular metastases by primary tumor source

| **Primary Tumor Source** | **Relative Frequency^a^** | **Age of Presentation** | **Sex Predisposition** | **Typical Clinical Features** | **Auxiliary Examination Findings** |
| --- | --- | --- | --- | --- | --- |
| **Breast carcinoma** [26,27] | Most common (40–50%) | Mean 60 years | Female predominance | Flat, placoid, creamy-yellow choroidal lesions; often multifocal & bilateral; subretinal fluid; long latency possible (years after diagnosis); strong association with bilateral involvement | Late hyperfluorescence on FA; echo-density with overlying subretinal fluid on US; irregular nodular surface with overlying fluid on OCT |
| **Lung carcinoma (adenocarcinoma, small cell)** [28,29] | Second most common (20–30%) | Mean 62 years | Male predominance | Rapidly progressive unilateral/bilateral (dome-shaped choroidal mass); may be initial presentation of occult cancer | Mottled hyperfluorescence on FA; echo-density with dependent subretinal fluid on US; irregular nodular surface with overlying fluid on OCT |
| **GI tract (colon, stomach)** [30,31] | Less common (5–10%) | 50–70 years | Male predominance | Proptosis; yellow-white choroidal mass due to lipofuscin (mimics primary choroidal melanoma); usually unilateral | Fluorescence leakage & low fluorescence on FA; orbital mass invading intra/extraconal spaces, medially shifting optic nerve; hyperdense, heterogeneous with focal enhancement on CT; metabolically active on PET-CT |
| **Prostate carcinoma** [32] | Rare (<5%) | Over 60 years | Male only | Proptosis, diplopia, orbital pain due to scirrhous bone metastases; enophthalmos from fibrosis; amelanotic choroidal lesions, often late-stage disease | Hypodense mass on CT; staging, recurrence, and treatment response assessed on ^68^Ga-PSMA PET/CT |
| **Renal cell carcinoma** [33] | Rare (<5%) | Mean 60 years | Male predominance | Dome-shaped choroidal mass; highly vascular lesions; hemorrhage possible; history of late metastasis (years after nephrectomy) | Hypodense mass with bone erosion on CT; contrast-enhanced T1WI with fat suppression on MRI; prominent “double circulation” on angiography |
| **Cutaneous melanoma** [34,35] | Uncommon | Predominantly elderly, all ages | No significant gender difference | Variably pigmented choroidal or conjunctival mass; clinically difficult to distinguish from primary uveal melanoma; history is key | Echodense mass on US; “lumpy bumpy” choroidal surface on OCT |
| **Thyroid carcinoma** [36,37] | Rare | Mean 57 years | Male predominance | Slowly progressive choroidal mass | Large exudative retinal detachment overlying a choroidal mass on fundoscopy |
| **Gynecologic malignancies (ovary, cervix, uterus)** [38] | Rare | Mean 48 years | Female only | Usually unilateral; associated with disseminated disease | Irregular, lobulated surface on OCT; ILM folds at macula on fundus examination; soft-tissue mass in extraconal compartment with intraconal extension on CT |
| **Hematologic malignancies (leukemia, lymphoma)** [39,40] | Variable | All ages | No significant gender difference | Retinal hemorrhages, infiltrates, optic disc edema | Inner retinal lesions throughout posterior pole on OCT; hyperechoic vitreous opacities with flat retina & no masses on US |
| **Head and neck cancers**[41,42] | Rare | Middle-aged & elderly | Male predominance | Proptosis, diplopia, orbital mass effect | Space-occupying lesion compressing optic nerve on MRI; exudative retinal & choroidal detachment on US |
| **Carcinoid tumors** [43] | Rare | Mean 53 years (range 20–80) | Possible male predominance | Classic yellow-white choroidal mass with overlying RPE mottling; often associated with chronic subretinal fluid (may mimic central serous retinopathy) | Amelanotic solid lesion with associated subretinal fluid on ophthalmoscopy |
| **Sarcomas** [44] | Very rare | Predominantly pediatric, also adults | No significant gender difference | Aggressive lesions; rapid progression | Well-defined isointense or dense mass with brain tissue on CT; isointense or slightly hypointense on T1WI & T2WI; homogeneous enhancement on enhanced scans |
| **Neuroblastoma (pediatric)** [45] | Most common orbital met in children | Predominantly middle-aged & elderly (note: pediatric tumor) | No significant gender difference | Acute, dramatic proptosis with periorbital ecchymosis (“raccoon eyes”); may cause opsoclonus-myoclonus syndrome | Homogeneous, isolated, ill-defined soft tissue density mass in orbit on CT |

OCT: optical coherence tomography; FA: fluorescein angiography; US: ultrasonography; CT: computed tomography; GI: gastrointestinal; ILM: internal limiting membrane; RPE: retinal pigment epithelium.

^a^ as cause of ocular metastasis.
